# Decision support framework for prioritizing labor protection measures to enhance workplace safety and compliance in Industry 4.0 environments

**DOI:** 10.3389/fpubh.2026.1781020

**Published:** 2026-02-24

**Authors:** Wenrui Lei

**Affiliations:** 1 Johns Hopkins University, Baltimore, MD, United States; 2 Anhui Bisheng Engineering Construction Company Limited, Hefei, Anhui, China

**Keywords:** Industry 4.0, labor protection measures, MARCOS method, multi-criteria decision-making (MCDM), occupational safety and health, sensitivity analysis

## Abstract

**Introduction:**

Ensuring adequate labor protection in Industry 4.0 environments has become increasingly complex due to the integration of automation, cyber–physical systems, smart sensors, and digital safety technologies. The selection of appropriate labor protection measures requires a systematic and transparent decision-support framework capable of handling multiple, often conflicting, evaluation criteria.

**Methods:**

This study proposes a comprehensive multi-criteria decision-making (MCDM) framework for prioritizing labor protection measures. Four objective weighting techniques, Entropy, Criteria Importance Through Intercriteria Correlation (CRITIC), Method based on the Removal Effects of Criteria (MEREC), and Criteria Impact Loss on System (CILOS), were integrated and fused using the Bonferroni aggregation operator. The Measurement of Alternatives and Ranking according to Compromise Solution (MARCOS) method was employed as the primary ranking approach. Comparative validation was performed using TOPSIS, VIKOR, EDAS, WASPAS, and PROMETHEE-II. Robustness assessment included rank correlation analysis (Kendall's tau and Spearman's rho), one-at-a-time (OAT) sensitivity analysis, consensus ranking through Borda and Copeland rules, and a Stability Index evaluation.

**Results:**

The results consistently identify Personal Protective Equipment (PPE) as the most preferred labor protection measure, achieving the highest MARCOS utility value (0.7124) and demonstrating strong robustness underweight perturbation scenarios. Administrative Controls ranked second, exhibiting exceptional stability across sensitivity analyses. Safety Training Programs demonstrated competitive performance but moderate sensitivity to variations in weight. High inter-method agreement was observed, with Kendall's tau (t) and Spearman's rho (r) values exceeding 0.80, confirming ranking consistency across MCDM techniques.

**Discussion:**

The proposed integrated MCDM framework provides a robust, objective, and reproducible approach for prioritizing labor protection measures in Industry 4.0 workplaces. By combining multiple objective weighting schemes with compromise-based ranking and comprehensive stability assessment, the model enhances decision transparency and reliability. The framework supports evidence-based formulation and strategic implementation of safety policies in digitally transformed industrial environments.

## Introduction

The transition toward Industry 4.0, characterized by cyber–physical systems, interconnected automation, data-driven operations, and human–technology collaboration, has fundamentally reshaped occupational safety and health (OSH). While digitalization improves productivity and process control, it also introduces emerging hazards related to human–robot interaction, complex assembly ecosystems, sensor-driven work practices, and technology-induced cognitive loads. Contemporary OSH scholarship increasingly frames this shift as a move toward human-centered socio-technical safety paradigms, where safety strategies must evolve beyond traditional compliance checklists toward integrated, multi-level deployment models aligned with sustainability and human wellbeing (1). In parallel, organizations are deploying Industry 4.0 technologies to strengthen OSH through real-time monitoring, proactive risk identification, and rapid corrective actions, but this introduces new decision complexities around feasibility, cost, acceptance, and long-term effectiveness (2). Recent research highlights that the transition from Industry 4.0 to Industry 5.0 is increasingly characterized by the integration of artificial intelligence with sustainability and human-centric industrial transformation, reshaping decision-making processes in digitally enabled workplaces (3). The growing adoption of artificial intelligence in organizational settings has been shown to drive sociotechnical transformation, emphasizing the need to balance technological advancement with human, organizational, and ethical considerations (4). Emerging evidence suggests that decision-making in advanced industrial contexts increasingly requires structured governance and auditing mechanisms to manage the complexity introduced by digital and human-centric Industry 5.0 paradigms (5).

In this study, the term “labor protection measures” is used as an inclusive concept to encompass the full spectrum of occupational safety interventions commonly described in the literature as safety measures or safety barriers. In line with international OSH frameworks such as ILO-OSH and ISO 45001, labor protection integrates both preventive objectives (hazard elimination and risk reduction at source) and protective objectives (mitigation of exposure and injury severity) [(6, 62, 63)]. The term does not imply a purely reactive, protection-oriented approach but reflects the combined prevention–protection logic embedded in the hierarchy of controls and contemporary socio-technical safety systems, particularly in Industry 4.0 environments.

A persistent concern across high-risk sectors is that workplace accidents remain costly and disruptive, affecting both schedule performance and organizational competitiveness. Empirical evidence from construction and infrastructure contexts shows that accident impacts are not uniform: sub-sector characteristics and organizational size influence the magnitude of cost overruns and schedule delays, strengthening the case for targeted, prioritized safety interventions rather than uniform “one-size-fits-all” measures (7). At the systems level, OSH management systems (OHSMS) are recognized as essential for sustainable safety improvements. Yet systematic reviews identify recurring barriers, including insufficient safety culture, weak communication, limited training, and inconsistent PPE use, factors that directly affect implementation success and the real-world performance of labor protection measures (8). These realities motivate a structured prioritization approach that reconciles multiple objectives and constraints while remaining transparent and defensible in Industry 4.0 environments.

Labor protection measures are commonly organized through the hierarchy of controls, spanning engineering controls, administrative controls, training, and personal protective equipment (PPE), which provides a structured approach for prioritizing hazard elimination and risk reduction at source before reliance on behavioral or protective measures (9–13). However, Industry 4.0 alters both the risk landscape and the intervention toolkit. For example, collaborative robots (co-bots) can reduce ergonomic risk and manual handling hazards when integrated appropriately into production operations, yielding measurable safety and productivity benefits in practical settings (14). Similarly, motion capture and virtual/immersive environments are being leveraged to redesign assembly systems with explicit inclusion of OSH considerations, demonstrating that digital design methods can embed safety early in workplace planning (15) and can be further supported by contemporary motion capture systems for ergonomic assessment and workplace optimization (16).

Training-related interventions also show rapid innovation. Reviews and empirical studies suggest that combining active training methods (discussion, gamification) with technology-enabled modalities (e.g., VR/AR/MR simulations) can strengthen engagement and improve safety learning outcomes (17). In construction safety contexts, experiential safety training has been shown to enhance risk perception and reduce tolerance for unsafe behaviors, an effect particularly valuable when “hidden” or less-obvious hazards are prevalent (18). Mixed reality is likewise being explored as a training tool for hazard recognition in assembly operations, offering practical advantages in delivering hands-on simulated experiences without real harm (19). These strands indicate that labor protection in Industry 4.0 must be evaluated not only for theoretical risk reduction but also for deployability, user acceptance, and training effectiveness.

Digital monitoring and data-driven safety management further extend labor protection strategies. Sensor fusion and deep learning approaches for worker activity recognition in IoT environments illustrate the feasibility of real-time monitoring frameworks that can support proactive safety actions (20). At the same time, evidence cautions against technological “solutionism”. Critical reviews of wearable sensor technologies for construction OSH highlight methodological limitations and the risk of premature adoption narratives, suggesting the need for careful prioritization and context-aware feasibility assessments before widespread deployment (21). These mixed findings reinforce the importance of a structured selection framework that weighs safety benefits against costs, complexity, and real-world readiness.

Selecting labor protection measures in Industry 4.0 environments is inherently multi-objective. Decision-makers must balance affordability and implementation feasibility with risk-reduction effectiveness, regulatory compliance, worker acceptance, and sustainability. While this study is explicitly situated within the context of Industry 4.0, it is important to acknowledge the growing discourse surrounding Industry 5.0. That emphasizes human-centricity, sustainability, and system resilience alongside technological advancement. Many of the evaluation dimensions considered in this study, including worker acceptance, regulatory compliance, sustainability, and long-term impact, are directly aligned with the core principles of Industry 5.0 (22, 23). Accordingly, the proposed decision-support framework can be viewed as forward-compatible with Industry 5.0 objectives. Even though the analytical focus remains on Industry 4.0 environments characterized by cyber–physical systems, automation, and data-driven operations. This positioning ensures conceptual continuity with emerging industrial paradigms while preserving a clear and well-defined decision context. The literature on safety decision-making increasingly emphasizes the role of multi-criteria decision-making (MCDM) to address such trade-offs. In construction safety, MCDM methods have been applied across diverse themes (risk assessment, safety programs, safety culture), and systematic reviews indicate that practitioners benefit from structured models when multiple criteria must be evaluated simultaneously (24). Beyond construction, multi-criteria models are also used to integrate OSH performance with productivity considerations, particularly relevant for socio-technical contexts such as aging workforces, demonstrating that safety decisions are seldom isolated from operational objectives (25).

A key methodological challenge is the weighting of criteria. Many safety studies depend heavily on subjective expert weighting, which can be inconsistent across contexts and challenging to reproduce. Consequently, engineering and safety evaluations increasingly adopt objective or combined weighting approaches. For instance, enterprise safety management systems have been evaluated using combination weighting structures that include Criteria Importance Through Intercriteria Correlation (CRITIC) within broader aggregation mechanisms, illustrating practical pathways for objective weighting to support safety evaluation (26). Similarly, safety evaluation in mining and infrastructure contexts has applied entropy-based and hybrid weighting strategies to improve reasonableness and reduce subjective distortion (27). In smart community safety research, CRITIC-based weighting combined with Technique for Order Preference by Similarity to Ideal Solution (TOPSIS) has demonstrated interpretable prioritization results that can guide targeted improvements (28). These studies collectively justify the use of objective weighting methods as an analytically rigorous foundation for prioritizing labor protection. Structured decision-support frameworks are increasingly adopted to overcome cognitive bias, habitual decision-making, and status quo inertia in complex managerial contexts (29).

Within the family of MCDM methods, Measurement of Alternatives and Ranking according to Compromise Solution (MARCOS) has gained traction due to its explicit benchmarking of alternatives against both ideal and anti-ideal reference solutions and its utility-based interpretation of performance. MARCOS has been applied successfully across decision domains where trade-offs are complex, and stakeholder accountability is important. Early applications include sustainable supplier selection and logistics/HR evaluation models, often validated through comparisons with established methods and sensitivity analyses (30–32). MARCOS has also been extended under uncertainty (e.g., intuitionistic fuzzy environments) and assessed via comprehensive sensitivity tests, reinforcing its suitability for real-world evaluations where decision inputs are imperfect (33). Hybridizations with other decision frameworks and gray systems further indicate MARCOS' compatibility with contexts requiring robust inference under data ambiguity (30, 34).

In safety-related environments, a benchmarking logic is particularly valuable: decision-makers often need to justify why a given safety measure is “closest to the ideal” under constrained budgets and operational limitations. Moreover, Occupational safety in Industry 4.0 environments decisions frequently involve technologies at different maturity levels (e.g., training simulations vs. wearables vs. co-bots), making a compromise-based, ideal/anti-ideal anchored approach appropriate. Nevertheless, despite the growing methodological maturity of MARCOS, its direct use for prioritizing labor protection measures specifically in Industry 4.0 environments remains limited in the current literature compared with more frequently deployed approaches such as TOPSIS, VlseKriterijumska Optimizacija I Kompromisno Resenje (VIKOR), and fuzzy AHP-based risk prioritization (35, 36). This creates a clear opportunity for methodological contribution and application-focused advancement.

A consistent theme in modern MCDM research is that single-method rankings can be sensitive to changes in weights and model assumptions; therefore, robustness validation has become a practical necessity. Sensitivity analysis is widely used in advanced MCDM applications to assess stability under perturbations and to determine whether top-ranked alternatives are resilient to plausible changes in preferences or information content (31–33). In safety decision-making, this is particularly important because safety priorities can shift due to regulatory updates, incident learnings, and constraints on technology adoption. Additionally, rank correlation measures (e.g., Kendall's τ and Spearman's ρ) provide quantitative evidence of agreement across MCDM methods, enabling decision-makers to interpret divergences as meaningful methodological differences rather than random inconsistency.

Consensus ranking approaches such as Borda and Copeland aggregation address another practical need: safety decisions in organizations often require collective justification across multiple analytical lenses and stakeholders. When combined with stability indicators derived from scenario-based sensitivity testing, consensus ranking can distinguish high-performing alternatives that are also robust under uncertainty. This is especially relevant for Occupational safety in Industry 4.0 environments, where technology readiness and workforce acceptance can materially change the feasibility and effectiveness of interventions (17, 21). Consensus aggregation techniques are widely employed to enhance the reliability and acceptability of MCDM outcomes in complex design and management problems (37).

The literature review identifies three critical gaps. First, Industry 4.0 introduces a diverse mix of labor protection measures ranging from conventional controls to digital monitoring and immersive training, yet practical prioritization tools remain fragmented across methods and domains (2, 15). Second, while objective weighting and combined weighting approaches are increasingly applied in safety and engineering evaluations, there remains a need for robust, transparent fusion of multiple objective weights within a single decision pipeline suitable for labor protection selection (26, 28). Third, many studies emphasize the importance of sensitivity testing and method comparison. Still, fewer integrate correlation, sensitivity, and consensus ranking into an end-to-end framework that produces a defensible final decision set for Industry 4.0 labor protection planning (24, 32). Accordingly, the present study is guided by the following research objectives:

- To identify and structure a comprehensive set of evaluation criteria for assessing labor protection measures in Industry 4.0 environments, reflecting economic, operational, regulatory, human-centric, and sustainability dimensions.
- To objectively determine and robustly fuse criteria weights using multiple objective weighting methods to minimize subjectivity and enhance the reliability of labor protection prioritization.
- To prioritize alternative labor protection measures using the MARCOS method by benchmarking their performance against ideal and anti-ideal reference solutions within an MCDM framework.
- To validate the robustness and consistency of the obtained rankings through comparative MCDM analysis, rank correlation measures, sensitivity testing, and consensus-based aggregation are supported by stability indices.

In response, the present study develops a unified decision-support framework that: (i) computes objective criteria weights through multiple objective methods and fuses them using a robust aggregation operator; ranks labor protection measures using MARCOS as a benchmarking-based method aligned with ideal and anti-ideal solutions; validates ranking reliability through comparative MCDM benchmarking and rank correlation analysis; and (iv) confirms robustness through structured sensitivity testing and final consensus ranking supported by stability indices. This integrated approach is designed to support practical decision-making in selecting labor protection strategies in Industry 4.0 environments. The framework is intended to support occupational safety decision-making by providing a transparent, validated, and robustness-aware prioritization process, rather than serving solely as a selection mechanism.

The remainder of this paper is organized as follows. Section 2 presents the methodological framework, covering the construction of the decision matrix, the weighting of objective criteria, the MARCOS ranking approach, and robustness validation techniques. Section 3 describes the selection of labor protection alternatives and evaluation criteria, along with the development of the decision matrix. Section 4 reports and discusses the results of the MCDM analysis, including comparative benchmarking, sensitivity analysis, and consensus ranking. Section 5 outlines the practical, policy, and methodological implications of the findings. Finally, Section 6 concludes the paper by summarizing key contributions, discussing limitations, and suggesting directions for future research.

## Methodology

**Figure 1:**
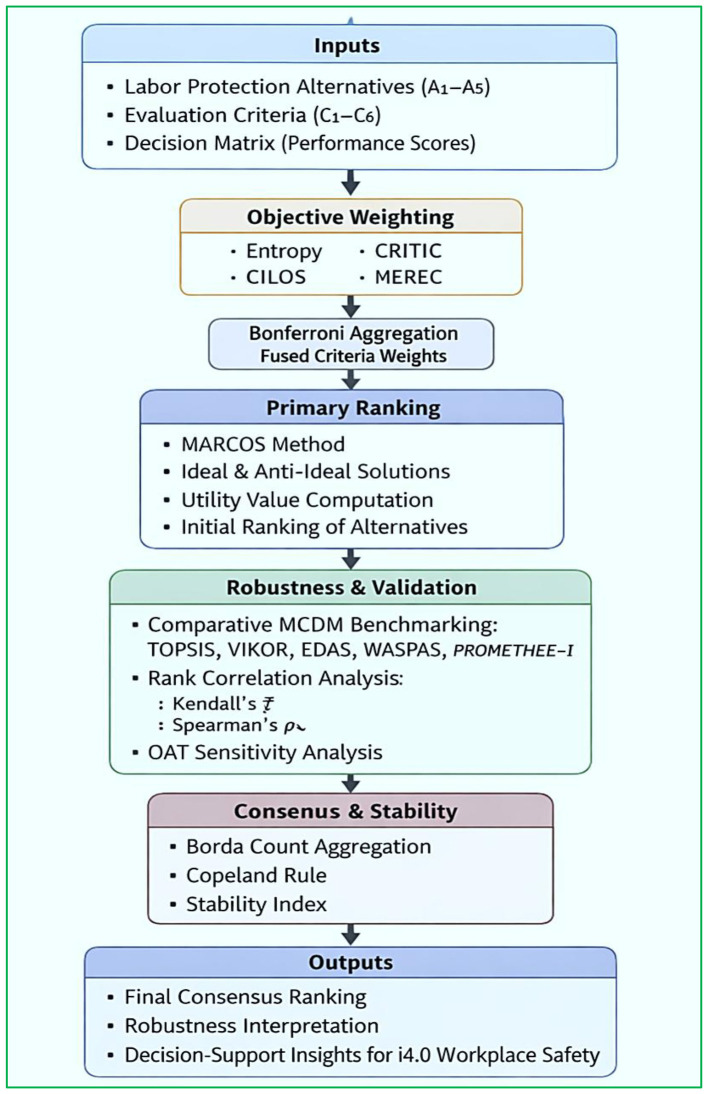
Overall methodological framework for prioritizing labor protection measures in Industry 4.0 environments (one-at-a-time: OAT, Industry 4.0: i4.0).

This study employs a structured, multi-stage decision-support methodology to prioritize labor protection measures in Industry 4.0 settings. The framework integrates objective criteria weighting, multiple MCDM techniques, robustness evaluation, and consensus-based aggregation to ensure reliability, transparency, and reproducibility. Figure 1 depicts the schematic overview of the proposed decision-support methodology for prioritizing labor protection measures in Industry 4.0 environments. The framework integrates objective criterion weighting, MARCOS-based ranking, comparative MCDM benchmarking, sensitivity analysis, and consensus aggregation to ensure robust, transparent decision-making.

### Step 1: construction of the decision matrix

The methodological process to prioritize labor protection measures in Industry 4.0 settings begins with the construction of a decision matrix *X* = [*x*_*ij*_]_*n*×*m*_, where *x*_*ij*_ denotes the performance of the alternative *A*_*i*_ (*i* = 1, 2, …, *n*) under criterion *G*_*j*_ (*j* = 1, 2, …, *m*). This matrix provides a unified quantitative representation that enables systematic comparison of all labor protection alternatives across all evaluation criteria.

Each criterion is first classified according to its optimization direction using a directional indicator δ_*j*_ as per Equation 1, which guides the choice of the appropriate normalization formula.


$$\begin{array}{l} \delta_{j}=\{\begin{array}{c} +1,\&if\;G_{j}\;is\;benefit-oriented\\ -1,\&if\;G_{j}\;is\;cost-oriented \end{array} \end{array}$$ (1)

In this study, all criteria are modeled as benefit-oriented, reflecting a safety-first perspective in which higher scores indicate better labor protection performance.

### Step-2: normalization and reference profiles

The labor protection criteria may differ in scale and magnitude; the original decision matrix is transformed into a dimensionless, comparable form through normalization, yielding the matrix *R* = [*r*_*ij*_]. For benefit-type criteria, the normalized value is obtained using Equation 2, whereas for cost-type criteria, it is obtained using Equation 3. It maps all entries to the interval [0, 1]. It also preserves preference orientation. Normalization is retained even when the original scores are defined on a uniform scale, ensuring consistency when benchmarking alternatives against ideal and anti-ideal reference solutions in the MARCOS procedure.


$$\begin{array}{l} r_{ij}=\frac{x_{ij}}{\underset{i}{\max}\;x_{ij}} \end{array}$$ (2)

$$\begin{array}{l} r_{ij}=\frac{\underset{i}{\min}\;x_{ij}}{x_{ij}} \end{array}$$ (3)

Ideal and anti-ideal reference profiles are then appended to the normalized matrix as defined by Equation 4. It presents the best attainable and worst observed performance across all criteria. These reference vectors serve as benchmarks for evaluating each alternative's relative position in the subsequent ranking phase.


$$\begin{array}{l} R^{+}=\left[\underset{i}{\max}\;r_{ij}\right],\\ R^{-}=\left[\underset{i}{\min}\;r_{ij}\right] \end{array}$$ (4)

### Step-3: objective determination of criteria weights

To eliminate subjectivity in assigning criterion weights, four objective weighting methods grounded in different theoretical principles are employed: Entropy, CRITIC, Method based on the Removal Effects of Criteria (MEREC), and Criteria Impact Loss on System (CILOS) (38). All weighting procedures are applied to the original decision matrix *X*, thereby preserving the data's intrinsic variability.

#### Entropy objective weight method

For the Entropy method (39), the normalized proportion for the criterion *G*_*j*_ is defined as in Equation 5, and the entropy value is given by Equation 6. The corresponding entropy-based weight is attained by Equation 7.


$$\begin{array}{l} q_{ij}=\frac{x_{ij}}{\sum_{i=1}^{n}x_{ij}} \end{array}$$ (5)

$$\begin{array}{l} E_{j}=-\kappa \overset{n}{\underset{i=1}{\sum }}q_{ij}\ln\left(q_{ij}\right),\kappa =\frac{1}{\ln\left(n\right)} \end{array}$$ (6)

$$\begin{array}{l} w_{j}^{\left(EN\right)}=\frac{1-E_{j}}{\sum_{j=1}^{m}\left(1-E_{j}\right)} \end{array}$$ (7)

#### CRITIC objective weight method

The CRITIC method combines the dispersion and conflict of each criterion (38). Let *G*_*j*_ denote the *j*-th criterion, and let *r*_*jk*_ = *corr*(*G*_*j*_, *G*_*k*_)be the correlation coefficient between criteria *G*_*j*_ and *G*_*k*_. The information content of the criterion *G*_*j*_ is computed as Equation 8.


$$\begin{array}{l} I_{j}=s_{j}\overset{m}{\underset{k=1}{\sum }}\left(1-\;\gamma_{jk}\right), \end{array}$$ (8)

here *s*_*j*_ is the standard deviation of the criterion *G*_*j*_ and *m* is the total number of criteria. A higher *I*_*j*_ indicates that the criterion has both greater variability and lower redundancy with other criteria, thus carrying more useful information for the decision problem.

The corresponding CRITIC weight for the criterion *G*_*j*_ is obtained by normalizing its information content across all criteria, as in Equation 9.


$$\begin{array}{l} w_{j}^{\left(CR\right)}=\frac{I_{j}}{\sum_{j=1}^{m}I_{j}} \end{array}$$ (9)

so that $\overset{m}{\underset{j=1}{\sum }}w_{j}^{CR}=1$ and $w_{j}^{CR}\geq 0$ for all *j*.

#### MEREC objective weight method

The MEREC method evaluates each criterion's importance by quantifying the performance loss resulting from its removal (38). The aggregate score of the alternative *A*_*i*_ with all criteria included, it is as per Equation 10. While ${P_{i}}^{-j}$ denotes the score obtained when criterion *G*_*j*_ is removed. The total removal effect of the criterion *G*_*j*_ is given by Equation 11 and the MEREC weight is given by Equation 12.


$$\begin{array}{l} P_{i}=\overset{m}{\underset{j=1}{\sum }}\omega_{j}\;r_{ij} \end{array}$$ (10)

$$\begin{array}{l} L_{j}=\overset{n}{\underset{i=1}{\sum }}|P_{i}-{P_{i}}^{-j}| \end{array}$$ (11)

$$\begin{array}{l} w_{j}^{\left(MR\right)}=\frac{L_{j}}{\sum_{j=1}^{m}L_{j}} \end{array}$$ (12)

#### CILOS objective weight method

The CILOS method assesses criterion importance as a function of its contribution to closing the performance gap relative to an ideal condition (38). The cumulative reciprocal measure is defined as in Equation 13 and with the associated CILOS weight in Equation 14.


$$\begin{array}{l} T_{j}=\overset{n}{\underset{i=1}{\sum }}\frac{1}{x_{ij}} \end{array}$$ (13)

$$\begin{array}{l} w_{j}^{\left(CI\right)}=\frac{T_{j}}{\sum_{j=1}^{m}T_{j}} \end{array}$$ (14)

### Step-4: fusion of objective weights via the bonferroni operator

To obtain a robust composite weight vector, the four objective weight sets are fused using a Bonferroni-type aggregation operator (40).

Let $w_{j}^{\left(k\right)}$ denote the weight of the criterion *G*_*j*_ obtained from the *k*-th objective method, with *k* = 1, 2, …, *K*. The fused weight ${\tilde{w}}_{j}$ is expressed as in Equation 15.


$$\begin{array}{l} {\tilde{w}}_{j}=\frac{1}{K}\overset{K}{\underset{k=1}{\sum }}\left(w_{j}^{\left(k\right)}+\frac{w_{j}^{\left(k\right)}w_{j}^{\left(L\right)}}{2}\right) \end{array}$$ (15)

where L denotes a paired method index within the Bonferroni formulation, followed by normalization of ${\tilde{w}}_{j}$ over all criteria. This operator captures interaction effects between weighting schemes and reduces the sensitivity of the final weights to outlier values produced by any single method.

### Step-5: MARCOS ranking procedure

The MARCOS method is employed as the primary MCDM ranking procedure, using the normalized matrix *R* and the fused weight vector ${\tilde{w}}_{j}$ (40, 41). First, the weighted normalized values are computed as in Equation 16, and the overall performance score for each alternative is calculated using Equation 17. Relative indicators are then defined as in Equation 18, and the final utility value of the alternative *A*_*i*_ is given by Equation 19.


$$\begin{array}{l} u_{ij}=r_{ij}\cdot {\tilde{w}}_{j} \end{array}$$ (16)

$$\begin{array}{l} Q_{i}=\overset{m}{\underset{j=1}{\sum }}u_{ij} \end{array}$$ (17)

$$\begin{array}{l} \lambda_{i}^{+}=\frac{Q_{i}}{Q^{+}},\\ \lambda_{i}^{-}=\frac{Q_{i}}{Q^{-}} \end{array}$$ (18)

$$\begin{array}{l} U_{i}=\frac{\lambda_{i}^{+}}{\lambda_{i}^{-}} \end{array}$$ (19)

### Step-6: comparative benchmarking with other MCDM methods

To validate the robustness of the MARCOS-based ranking, comparative analyses are conducted using other well-established MCDM methods, namely VIKOR, Preference Ranking Organization Method for Enrichment Evaluation II (PROMETHEE-II), TOPSIS, Evaluation based on Distance from Average Solution (EDAS), and Weighted Aggregated Sum Product Assessment (WASPAS), all applied with the same fused weights ${\tilde{w}}_{j}$.

VIKOR is used for compromise-based ranking. VIKOR focuses on identifying an alternative that provides the closest balance between group utility and individual regret. Its results differ because it emphasizes reaching a “compromise solution” rather than simply minimizing distance (42).

PROMETHEE-II is based on outranking logic; it evaluates pairwise preferences using preference functions and provides a complete ranking via net flow values. Its impact varies as it captures preference intensity rather than pure distance or aggregation (43).

TOPSIS, a distance-based technique, ranks alternatives relative to the ideal and negative-ideal solutions. Its results differ since it prioritizes closeness to the best scenario and remoteness from the worst (44).

EDAS, this deviation-based method measures how far each alternative's performance deviates from the average solution. The variation arises because it centers decisions on positive and negative distances from the mean rather than on absolute ideal points (40, 45).

WASPAS combines weighted-sum and weighted-product models to aggregate criterion scores. Its differing impact comes from integrating both additive and multiplicative principles, improving ranking stability and sensitivity (42, 46).

### Step-7: rank correlation and sensitivity analysis

To assess the consistency of alternative rankings across different MCDM methods, non-parametric rank correlation measures are employed (47).

Kendall's tau is given by Equation 20.


$$\begin{array}{l} \tau =\frac{N_{c}-N_{d}}{\frac{1}{2}n\;\left(n-1\right)} \end{array}$$ (20)

where *N*_*c*_ and *N*_*d*_ are the numbers of concordant and discordant pairs of alternatives, respectively.

Spearman's rank correlation coefficient between rankings *a* and *b* is computed as with Equation 21.


$$\begin{array}{l} \rho =1-\frac{6\sum_{i=1}^{n}{\left(r_{i}^{\left(a\right)}-r_{i}^{\left(b\right)}\right)}^{2}}{n\left(n^{2}-1\right)} \end{array}$$ (21)

where $r_{i}^{\left(a\right)}$ and $r_{i}^{\left(b\right)}$ denote the positions of the alternative *A*_*i*_ in rankings *a* and *b*.

### Sensitivity analysis

Sensitivity analysis is conducted to examine the stability of rankings under perturbations of the criteria weights (47). For a selected criterion *G*_*h*_, the adjusted weights are defined as


$$\begin{array}{l} \omega_{h}^{\prime }=\omega_{h}+\epsilon ,\\ \omega_{j}^{\prime }=\omega_{j}-\frac{\epsilon }{m-1}\left(j\neq h\right) \end{array}$$ (22)

where ϵ is a small perturbation parameter. The utility scores and rankings are recomputed under these modified weights to observe how changes in the importance of individual criteria affect the prioritization of alternatives.

### Step-8: consensus ranking and stability index

To obtain a final consensus ranking that integrates information from all MCDM methods and sensitivity scenarios, Borda count and Copeland aggregation rules are applied. These procedures combine multiple rank orders into a single collective ranking that reflects the overall preference structure (47).

#### Borda count formulation

Assume there are *n* alternatives *A*_1_, *A*_2_, …, *A*_*n*_ and *M* ranking sources (e.g., different MCDM methods or scenarios). Let $r_{i}^{\left(m\right)}$ be the rank position of the alternative *A*_*i*_ in ranking *m*, where $r_{i}^{\left(m\right)}\in \left\{1,2,\ldots ,n\right\}$ and smaller values denote better ranks. The Borda score of *A*_*i*_ in ranking *m* is commonly defined by Equation 23 and the total Borda score of the alternative *A*_*i*_ across all rankings is given by Equation 24. Alternatives are then ordered in descending order of *B*_*i*_; higher *B*_*i*_ indicates a more preferred alternative in the collective Borda ranking.


$$\begin{array}{l} B_{i}^{\left(m\right)}=n-r_{i}^{\left(m\right)} \end{array}$$ (23)

$$\begin{array}{l} B_{i}=\overset{M}{\underset{m=1}{\sum }}B_{i}^{\left(m\right)}=\overset{M}{\underset{m=1}{\sum }}\left(n-r_{i}^{\left(m\right)}\right) \end{array}$$ (24)

#### Copeland rule formulation

The Copeland method is based on pairwise comparisons of alternatives across all rankings (47). For each ordered pair of distinct alternatives (*A*_*i*_, *A*_*j*_), define: *V*_*ij*_: number of rankings in which *A*_*i*_ is ranked higher (better) than *A*_*j*_. *V*_*ji*_: number of rankings in which *A*_*j*_ is ranked higher than *A*_*i*_. A pairwise preference indicator can be defined as Equation 25 and the Copeland score of alternative *A*_*i*_ is then by Equation 26. Alternatives are ranked in descending order of *C*_*i*_; a larger Copeland score *C*_*i*_ indicates that the alternative wins more pairwise contests (or loses fewer) against its competitors in the aggregated preference structure.


$$\begin{array}{l} C_{ij}=\{\begin{array}{cc} 1, & if\;V_{ij}>V_{ji}\left(A_{i}\text{ beats }A_{j}\right)\\ 0, & if\;V_{ij}=V_{ji}\left(\text{tie}\right)\\ -1, & if\;V_{ij}<V_{ji}\left(A_{i}\text{ loses }to\;A_{j}\right) \end{array} \end{array}$$ (25)

$$\begin{array}{l} C_{i}=\overset{n}{\underset{\begin{array}{c} j=1\\ j\neq i \end{array}}{\sum }}C_{ij} \end{array}$$ (26)

#### Stability index *SI*_*i*_

A Stability Index is defined for each alternative *A*_*i*_ as by Equation 27 (47). where “evaluation scenarios” encompass different methods, weighting schemes, and perturbation levels. The *SI*_*i*_ positions and provides a concise measure of ranking robustness across the entire decision-support framework.


$$\begin{array}{l} SI_{i}=\frac{Number\;of\;appearances\;of\;A_{i}\;in\;Top-k}{Total\;evaluation\;scenarios} \end{array}$$ (27)

## Labor protection measures: study design, data specification, and construction of the decision matrix

In this study, the hierarchy of controls is employed as a conceptual foundation rather than a rigid prescriptive ordering. While the traditional hierarchy prioritizes elimination and substitution, these measures are often constrained in Industry 4.0 environments by legacy production systems, high capital intensity, and limited flexibility for fundamental process redesign. Accordingly, an adapted hierarchy aligned with contemporary industrial contexts is adopted, focusing on intervention domains that are practically actionable within digitally enabled workplaces. This approach is consistent with recent occupational safety literature, which emphasizes contextualized and system-level safety strategies rather than strict adherence to idealized control sequences.

The alternatives considered in this study are intentionally defined as high-level labor protection intervention categories rather than individual Industry 4.0 technologies. This design choice reflects the strategic perspective of organizational occupational safety decision-makers, who typically evaluate and prioritize safety investments at the level of intervention domains (e.g., engineering controls, administrative controls, or digital monitoring systems) rather than selecting specific technologies in isolation. Adopting broader categories enables generalizable insights across sectors and avoids fragmenting the decision model into technology-specific cases that may rapidly evolve.

### Selection of labor protection alternatives

#### A1: engineering controls

Engineering controls represent the highest-priority hazard intervention strategy in the OSHA and ILO Hierarchy of Controls. These measures aim to eliminate or physically isolate hazards by modifying equipment, processes, or the work environment, e.g., machine guarding, noise barriers, ergonomic redesign, automation of hazardous tasks, and installation of ventilation or containment systems (11). Engineering controls are preferred because they reduce risk at the source without relying on worker behavior or compliance (9). As industrial processes evolve under Industry 4.0, engineering controls increasingly incorporate cyber-physical systems, automated shut-off mechanisms, and robotics-based hazard prevention (48, 49).

#### A2: administrative controls

Administrative controls consist of policies, procedures, and organizational strategies aimed at reducing workers' exposure to hazards by changing how work is scheduled, performed, or supervised. Examples include job rotation, shift scheduling, safe work procedures (SOPs), permit-to-work systems, and regular workplace inspections. These controls are widely endorsed by OSHA, ISO 45001, and national OSH authorities because they provide structured behavioral and procedural guidance in environments where hazards cannot be eliminated through engineering controls (12). Administrative controls remain vital in Industry 4.0 operations, especially where workers interact with automated machinery, digital systems, and new work patterns introduced by smart manufacturing. They help ensure consistent compliance, operational stability, and the institutionalization of safety practices throughout the organization (10, 13).

#### A3: personal protective equipment (PPE)

PPE acts as the last line of defense between workers and workplace hazards when engineering and administrative controls cannot fully eliminate risks. PPE includes helmets, gloves, respirators, safety goggles, protective footwear, hearing protection, and high-visibility clothing. OSHA and the ILO require that PPE be provided and used properly where needed, making it a legally essential part of workplace safety. In Industry 4.0 settings, PPE is increasingly enhanced with digital features like smart helmets with sensors, location-tracking wearables, biometric monitors, and connected safety devices. Although PPE relies on user compliance and does not remove hazards at their source, it remains a vital safety measure for preventing injuries during dangerous tasks (50, 51).

#### A4: safety training programs

Safety training is a fundamental part of international OSH management systems because it provides workers with the knowledge, skills, and behaviors needed to work safely and respond appropriately to hazards. ISO 45001 emphasizes the importance of developing competence, while OSHA requires training for various hazardous activities. Training programs might include hazard awareness, equipment handling, emergency response, ergonomics, safe practices for human–robot collaboration, and instructions on digital systems used in Industry 4.0 workplaces (52). Hands-on training enhances worker awareness, encourages safe habits, improves compliance, and decreases human error, one of the leading causes of industrial accidents (53). As industries adopt automation, robotics, and AI-based technologies, training that promotes digital literacy and safe interaction with machines becomes increasingly crucial for protecting workers (8; Occupational & Health).

#### A5: digital safety monitoring systems

Digital safety monitoring systems represent the latest development in workplace hazard management, driven by Industry 4.0 technologies. These systems leverage sensors, Internet of Things (IoT) devices, wearables, machine vision, cyber-physical systems, and artificial intelligence to continuously track environmental and operational conditions. They deliver real-time alerts, predictive risk analytics, automated safety responses, and data-driven insights that significantly improve hazard prevention and workplace responsiveness (54). Examples include gas detection networks, smart PPE, proximity detectors, fatigue monitoring devices, and safety dashboards connected to factory networks (55). EU-OSHA describes digitalization as a transformative force for future occupational safety, enabling proactive, high-precision monitoring that was previously impossible in traditional settings (56). By adopting digital safety systems, organizations enhance situational awareness, reduce dependence on manual inspections, and enable predictive maintenance to prevent accidents before they happen (57, 58).

The selected labor protection alternatives are defined as functionally distinct intervention domains rather than overlapping technical measures. Although safety training programs are often classified under administrative controls in conventional taxonomies, training is treated as a separate alternative in this study because it represents a dedicated human-centric intervention mechanism with distinct objectives, implementation dynamics, and performance implications in digitally complex work environments. Separating training enables a more granular assessment of its contribution relative to organizational policies and procedural controls.

Similarly, digital safety monitoring systems differ from engineering controls and personal protective equipment in their primary function. Engineering controls in this study refer to physical or technical modifications that isolate hazards at source, while personal protective equipment focuses on mitigating exposure at the individual level. In contrast, digital safety monitoring systems function as information-centric, decision-support mechanisms that leverage sensors, data analytics, and real-time feedback to enhance situational awareness and proactive risk management. To avoid double-counting, smart PPE elements are conceptually assigned to the digital monitoring alternative only insofar as their monitoring and data-generation functions are considered, while their protective function remains associated with PPE.

### Selection of evaluation criteria

The evaluation criteria were defined using a structured, multi-stage process. First, a targeted review of occupational safety and health literature and international regulatory frameworks (e.g., ILO-OSH, ISO 45001, EU-OSHA guidelines) was conducted to identify commonly used dimensions for assessing workplace safety interventions (9, 52–54, 56). This review highlighted recurring evaluation themes related to economic feasibility, operational effectiveness, regulatory compliance, human-centric considerations, and sustainability, which are particularly relevant in Industry 4.0 environments characterized by digitalization and human–technology interaction (55). Second, the preliminary set of criteria was refined through expert consultation to ensure contextual relevance and practical applicability (53). The expert panel comprised professionals with experience in occupational safety management, industrial engineering, and digital safety systems. Experts assessed the clarity, completeness, and non-redundancy of the proposed criteria and confirmed their suitability for evaluating labor protection measures in digitally enabled workplaces. Based on this process, six evaluation criteria were finalized for subsequent analysis. This combined literature-informed and expert-validated approach ensures that the selected criteria are theoretically grounded, practically meaningful, and aligned with contemporary occupational safety decision-making needs.

#### C1: life cycle cost (affordability)

C1 represents the life-cycle cost, encompassing both initial setup costs for equipment acquisition and installation and ongoing operational costs (maintenance, training, and system updates) associated with each labor protection measure. Life Cycle Costs are a key factor in evaluation, as financial feasibility greatly influences the adoption and sustainability of labor protection measures. Organizations must consider both initial setup expenses, such as buying equipment, installing engineering controls, or integrating digital monitoring systems, and ongoing operational costs, including maintenance, training, and regular replacement of safety components. Cost assessment is also necessary during the planning and budgeting phases of the OSHA Safety and Health Program Guidelines, which emphasize the importance of allocating sufficient resources for effective hazard control. The significance of affordability becomes even more critical in Industry 4.0 environments, where new technologies often require capital investments, system upgrades, and compatibility with existing infrastructure. Therefore, cost evaluation helps organizations balance safety performance with budget constraints, ensuring that the chosen measures are both practical and economically viable (13). C1 captures long-term economic feasibility rather than short-term expenditure, consistent with safety investment decision-making practices. The study does not exclude economic considerations; rather, it avoids a cost-minimization-dominated decision logic. C1 is included to capture long-term affordability, operational sustainability, and resource commitment. This approach reflects real-world occupational safety planning, where low-cost solutions may be unsustainable or ineffective if long-term maintenance, compliance, or human-centric factors are overlooked.

#### C2: risk-reduction effectiveness

Risk-reduction effectiveness measures how well a labor protection measure reduces the likelihood and severity of workplace accidents, occupational injuries, and near-miss events. This criterion aligns closely with the primary goal of OSH frameworks, which focus on interventions that eliminate hazards or significantly lower exposure. OSHA and ILO guidelines stress the importance of assessing controls using empirical data such as incident rates, hazard exposure levels, and past injury statistics. For instance, engineering controls typically offer high risk-reduction potential by removing hazards at their source, whereas PPE, though essential, usually provides lower and more variable levels of protection. In smart factories and Industry 4.0 work systems, digital monitoring tools further improve risk mitigation by enabling real-time hazard detection and predictive analytics (12, 50).

#### C3: regulatory compliance level

Regulatory compliance level is a key criterion because organizations must comply with local labor laws, national safety standards, ISO 45001 requirements, and international labor conventions. Compliance ensures that protective measures meet mandatory safety standards, including hazard control procedures, PPE certification, training requirements, and reporting obligations. ISO 45001 requires organizations to assess how well their controls meet legal and regulatory expectations, making compliance a vital part of operational planning and continuous improvement. Choosing measures that improve compliance helps reduce the risk of legal penalties, operational shutdowns, and reputational damage, while encouraging a culture of accountability and safety governance. In an Industry 4.0 environment, compliance also covers digital system security, data management, and the safe use of cyber-physical technologies (10, 59).

#### C4: worker acceptance and usability

Worker acceptance and usability reflect the extent to which a labor protection measure is perceived as acceptable, intuitive, and practical in daily operations. In Industry 4.0 environments, usability and acceptance are strongly interdependent: systems that are difficult to use are unlikely to be accepted, while poorly accepted measures are often underutilized despite their technical effectiveness. For this reason, these aspects are treated as a single, integrated criterion to capture their combined influence on real-world adoption and sustained compliance, while avoiding redundancy and double-counting within the decision model (50, 60).

#### C5: sustainability and long-term impact

Sustainability and long-term impact are defined using an integrated perspective consistent with the triple bottom line framework, encompassing environmental, economic, and social sustainability dimensions. From an environmental perspective, the criterion considers the potential of labor protection measures to reduce waste, emissions, or resource inefficiencies over time. From an economic perspective, it reflects long-term cost-effectiveness, durability, and maintenance implications beyond initial implementation. From a social perspective, it captures sustained worker wellbeing, reinforced safety culture, and long-term organizational resilience. These dimensions are assessed collectively to reflect the systemic and long-term nature of safety interventions in Industry 4.0 environments (61).

#### C6: ease of implementation

Ease of implementation measures how smoothly a labor protection intervention can be deployed within existing operational, technological, and organizational structures. ISO 45001 and OSHA guidelines require organizations to assess feasibility, resource availability, workforce capability, and the potential disruption associated with adopting new controls. This criterion covers installation complexity, training requirements, compatibility with Industry 4.0 systems, and the time required to achieve full operational effectiveness. In smart factories, ease of implementation becomes especially important because digital safety technologies often require integration with networks, sensors, robotics, and data platforms. Evaluating this criterion helps organizations choose measures that can be implemented efficiently without significant downtime or structural changes (10, 13).

### Rationale for benefit-type criteria and cost affordability

All selected criteria are modeled as benefit-type, including Life Cycle Cost (C1), ensuring that higher values consistently represent better performance across effectiveness, regulatory compliance, usability, sustainability, and ease of implementation. This design aligns the evaluation framework with safety-first principles, in which improvements in these dimensions directly enhance labor protection outcomes rather than entail trade-offs against safety. In international OSH guidance (e.g., ILO-OSH, ISO 45001, OSHA), core safety measures are framed as mandatory obligations rather than discretionary investments, which means they should not be systematically deprioritized solely on the basis of cost. In this study, cost minimization is therefore not treated as the dominant decision objective in order to avoid biasing the ranking toward purely economic considerations at the expense of safety performance. At the same time, long-term economic feasibility remains an essential component of occupational safety planning, particularly in Industry 4.0 environments where technologies differ in acquisition, operation, and maintenance demands. To reflect this, Life Cycle Cost (C1) is incorporated as a benefit-type criterion that captures affordability and long-term budgetary impact, with higher scores indicating more economically sustainable options. In this way, economic considerations are integrated into a balanced, multi-objective decision-support framework without undermining the primacy of safety-related benefits. In this way, economic considerations are integrated into a balanced, multi-objective decision-support framework while preserving the primacy of safety-related benefits in the final prioritization.

The performance scores used to construct the decision matrix (Table 1) were obtained through a structured expert-based evaluation supported by secondary sources. First, a preliminary assessment of labor protection measures was informed by relevant occupational safety standards, regulatory guidelines, and prior empirical studies to ensure baseline consistency across criteria. Subsequently, expert judgment was employed to evaluate the relative performance of each alternative with respect to each criterion, reflecting practical implementation conditions in Industry 4.0 environments. An expert panel comprising 7 professionals with backgrounds in occupational safety management, industrial engineering, and digital safety systems participated in the evaluation process. All experts had at least 8 years of professional experience in workplace safety–related roles. Experts independently scored each alternative against each criterion using a nine-point Likert scale (1 = very low performance, 9 = very high performance). To reduce individual bias, the final performance score for each alternative–criterion pair was obtained by averaging individual expert scores. The resulting aggregated scores were subsequently normalized within the MCDM framework prior to weighting and ranking. This approach is consistent with established expert-based MCDM practice and enables transparent, reproducible construction of the decision matrix while accommodating qualitative and quantitative safety considerations. Further details on the expert-based performance scoring process, including the scoring scale and an example evaluation template, are provided in Appendix A (Supplementary material).

**Table 1:** Decision matrix: labor protection measures to enhance workplace safety and compliance in Industry 4.0 environments.

**Alternative ↓/Criterion →**	**C1**	**C2**	**C3**	**C4**	**C5**	**C6**
	**Life cycle cost (affordability)**	**Risk-reduction effectiveness**	**Regulatory compliance level**	**Worker acceptance and usability**	**Sustainability and long-term impact**	**Ease of implementation**
A1 engineering controls	4	9	8	7	8	5
A2 administrative controls	8	7	7	8	6	8
A3 personal protective equipment	9	6	6	9	5	9
A4 safety training	7	8	7	8	7	7
A5 digital safety monitoring systems	3	9	9	6	9	6

The number of evaluation criteria was intentionally limited to six to balance analytical completeness with model parsimony. In MCDM, an excessive number of criteria may introduce redundancy, increase subjectivity in expert scoring, and reduce the interpretability of results. The selected criteria collectively capture the principal dimensions that govern occupational safety decision-making in Industry 4.0 environments, namely economic feasibility, operational effectiveness, regulatory compliance, human-centric adoption, sustainability, and implementation feasibility. Additional technology-specific attributes, such as technological maturity, scalability, and interoperability, were not introduced as independent criteria because their effects are implicitly reflected within implementation feasibility and sustainability considerations. Low technological maturity or poor interoperability directly constrains implementation feasibility, while limited scalability influences long-term sustainability and organizational resilience. Treating these attributes as separate criteria would therefore risk conceptual overlap and double-counting.

## Results and discussion

### Criteria for labor protection measures: objective weights calculation

Four complementary objective weighting methods, namely Entropy, CRITIC, MEREC, and CILOS, as formulated in Equations 5–14, are used to determine the relative importance of the six evaluation criteria. Each method captures a distinct aspect of criterion significance: Entropy reflects information dispersion, CRITIC incorporates variability and inter-criterion conflict, MEREC quantifies performance loss from criterion removal, and CILOS evaluates the contribution to closing the performance gap relative to ideal conditions. To mitigate bias associated with any single method and enhance robustness, the individual weight vectors were aggregated using the Bonferroni fusion operator, as defined in Equation 15.

Table 2 depicts the individual objective weights obtained from each method along with the final Bonferroni-fused weights. The results indicate that C1 (Life Cycle Cost/Affordability) receives the highest composite weight (0.3004). It highlights its dominant influence on decision outcomes even within a safety-first framework. It also replicates the substantial dispersion and discriminative power of cost-related performance scores across the alternatives under Entropy and CRITIC formulations. The second tier of importance comprises C6 (Ease of Implementation) and C5 (Sustainability and Long-Term Impact), with fused weights of 0.1583 and 0.1579, respectively. These criteria capture operational feasibility and long-term system resilience, both of which are critical in Industry 4.0 situations characterized by complex cyber–physical integration and sustainability-driven policy pressures. The near-equal weighting of these criteria suggests a balanced emphasis on practical deployability and enduring safety benefits. The criteria C2 (Risk-Reduction Effectiveness), C4 (Worker Acceptance and Usability), and C3 (Regulatory Compliance Level) exhibit moderately lower but comparable fused weights (0.1335, 0.1252, and 0.1247, respectively). This distribution indicates that, while these dimensions remain essential to occupational safety decision-making, their relative discriminative impact across the considered alternatives is less pronounced than that of cost, sustainability, and implementation feasibility. The close clustering of weights among C2, C3, and C4 suggests that the framework does not over-prioritize any single behavioral or regulatory dimension, thereby preserving multi-dimensional balance. The Bonferroni-fused weight vector demonstrates stability and methodological coherence by integrating diverse objective perspectives and preventing dominance by extreme values from any individual method. These fused weights are then used in the MARCOS ranking procedure (Equations 16–19) to ensure that alternative prioritizations reflect a robust, data-driven assessment of labor protection performance.

**Table 2:** Objective and Bonferroni-fused weights for labor protection measures.

**Criteria**	**Entropy**	**CRITIC**	**MEREC**	**CILOS**	**Bonferroni-fused weights**
C1	0.5110	0.2790	0.1442	0.2153	0.3004
C2	0.0785	0.1391	0.1814	0.1469	0.1335
C3	0.0647	0.1211	0.1721	0.1540	0.1247
C4	0.0628	0.1244	0.1767	0.1500	0.1252
C5	0.1415	0.1675	0.1628	0.1668	0.1579
C6	0.1415	0.1689	0.1628	0.1668	0.1583

**Figure 2:**
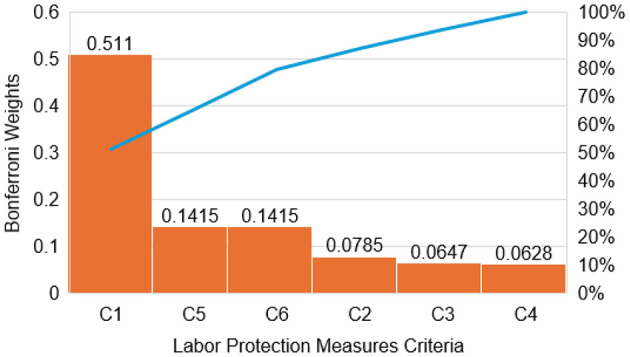
Pareto chart of Bonferroni-fused criteria weights highlighting dominant evaluation criteria in the prioritization of labor protection measures for Industry 4.0 environments.

Figure 2 utilized to highlight the relative dominance of evaluation criteria with the Bonferroni-fused weights using a Pareto chart. It enables a cumulative assessment of criterion importance by ranking the fused weights in descending order and illustrating their collective contribution to the overall decision structure. Life Cycle Cost (C1) alone accounts for nearly one-third of the total decision weight, confirming its dominant role in the objective weighting framework. When combined with Ease of Implementation (C6) and Sustainability and Long-Term Impact (C5), the cumulative contribution exceeds 60% of the total weight, indicating that these three criteria form the core of critical decision-making in Industry 4.0 labor protection planning. The Risk-Reduction Effectiveness (C2), Worker Acceptance and Usability (C4), and Regulatory Compliance Level (C3) contribute a smaller yet non-negligible share, reinforcing the multidimensional nature of occupational safety assessment without disproportionately amplifying behavioral or regulatory factors. This confirms that the fused weighting scheme successfully differentiates between high-impact and supporting criteria while preserving balance across economic, operational, and safety-oriented dimensions.

The high weight assigned to C1 indicates the importance of long-term economic feasibility in sustaining safety interventions, rather than a preference for low-cost solutions at the expense of effectiveness or worker wellbeing. The dominance of C1 in the weighting results reflects expert concern for the long-term economic sustainability of safety interventions rather than a preference for low-cost solutions, emphasizing the importance of balancing economic feasibility with protection effectiveness.

### Prioritizing labor protection measures in Industry 4.0 environments: benchmark by MARCOS

Labor protection measures were prioritized using the MARCOS method, employing the Bonferroni-fused objective weights derived in Section 4.1. The MARCOS approach evaluates each alternative relative to both ideal and anti-ideal reference solutions. It enables a transparent assessment of performance proximity under a multi-criteria safety framework.

Table 3 depicts the extended normalized decision matrix. It is constructed by joining the ideal and anti-ideal reference profiles to the set of alternatives. This step establishes the upper and lower performance bounds for each criterion, ensuring that all labor protection measures are evaluated within a unified comparative space. The normalized values indicate that A3 PPE and A5 (Digital Safety Monitoring Systems) achieve several criterion-wise maxima, whereas A1 (Engineering Controls) exhibits comparatively lower normalized values for cost and ease of implementation. The weighted normalized matrix is obtained using Equation 16 and reflects the combined influence of performance scores and Bonferroni-fused criteria weights. A3 achieves the highest weighted contributions for C1 (Life Cycle Cost), C4 (Worker Acceptance and Usability), and C6 (Ease of Implementation), indicating a strong balance between affordability, usability, and deployability. A5 shows dominant weighted values in C3 (Regulatory Compliance Level) and C5 (Sustainability and Long-Term Impact), highlighting its technological and long-term strategic advantages, albeit with moderate implementation feasibility. The relative closeness of each alternative to the ideal and anti-ideal solutions is quantified through the relative indicators $\lambda_{i}^{+}$ and $\lambda_{i}^{-}$, as defined in Equation 18. These indicators are subsequently integrated to compute the final utility value *U*_*i*_ using Equation 19.

**Table 3:** Prioritization of labor protection measures in Industry 4.0 environments based on MARCOS.

**Alternatives**	**C1**	**C2**	**C3**	**C4**	**C5**	**C6**
**Extended normalized matrix**						
Anti-ideal	0.3333	0.6667	0.6667	0.6667	0.5556	0.5556
A1	0.4444	1.0000	0.8889	0.7778	0.8889	0.5556
A2	0.8889	0.7778	0.7778	0.8889	0.6667	0.8889
A3	1.0000	0.6667	0.6667	1.0000	0.5556	1.0000
A4	0.7778	0.8889	0.7778	0.8889	0.7778	0.7778
A5	0.3333	1.0000	1.0000	0.6667	1.0000	0.6667
Ideal	1.0000	1.0000	1.0000	1.0000	1.0000	1.0000
**Weighted normalized matrix**						
Anti-ideal	0.1001	0.0890	0.0831	0.0835	0.0877	0.0879
A1	0.1335	0.1335	0.1108	0.0974	0.1404	0.0879
A2	0.2670	0.1039	0.0970	0.1113	0.1053	0.1407
A3	0.3004	0.0890	0.0831	0.1252	0.0877	0.1583
A4	0.2336	0.1187	0.0970	0.1113	0.1228	0.1231
A5	0.1001	0.1335	0.1247	0.0835	0.1579	0.1055
Ideal	0.3004	0.1335	0.1247	0.1252	0.1579	0.1583
	**Relative indicator** $\lambda_{\text{i}}^{+}$	**Relative indicator** $\lambda_{\text{i}}^{-}$	**Final utility** **U**_**i**_	**Ranks**		
Anti-ideal	0.5314	1.0000	0.4487			
A1	0.7036	1.3239	0.5940	5		
A2	0.8251	1.5526	0.6966	2		
A3	0.8437	1.5877	0.7124	1		
A4	0.8065	1.5177	0.6810	3		
A5	0.7053	1.3271	0.5955	4		
Ideal	1.0000	1.8818	0.8443			

The final utility values indicate that A3 PPE is the most preferred labor protection measure, with the highest utility score of *U*_*i*_ = 0.7124. This result reflects PPE's strong performance across cost efficiency, worker acceptance, and implementation simplicity, which are particularly critical in Industry 4.0 environments with diverse operational constraints. A2 (Administrative Controls) ranks second (*U*_*i*_ = 0.6966), demonstrating stable and consistent performance across most criteria, especially in terms of affordability and regulatory alignment. A4 (Safety Training Programs) occupies the third position (*U*_*i*_ = 0.6810), supported by balanced scores across all dimensions but lacking dominance in any single high-weight criterion. A5 (Digital Safety Monitoring Systems) is ranked fourth (*U*_*i*_ = 0.5955), despite excelling in sustainability and compliance, due to comparatively lower performance in cost and ease of implementation. A1 (Engineering Controls) ranks fifth (*U*_*i*_ = 0.5940), indicating that although structurally effective, such controls are less competitive when evaluated against modern Industry 4.0 constraints related to flexibility, affordability, and rapid deployment. The MARCOS-based prioritization demonstrates a transparent and interpretable ranking structure driven by proximity to ideal safety performance. The results confirm that labor protection strategies emphasizing usability, implementation feasibility, and balanced cost–performance trade-offs are most suitable for contemporary Industry 4.0 industrial environments.

### Comparative rankings of labor protection measures and correlation analysis

**Figure 3:**
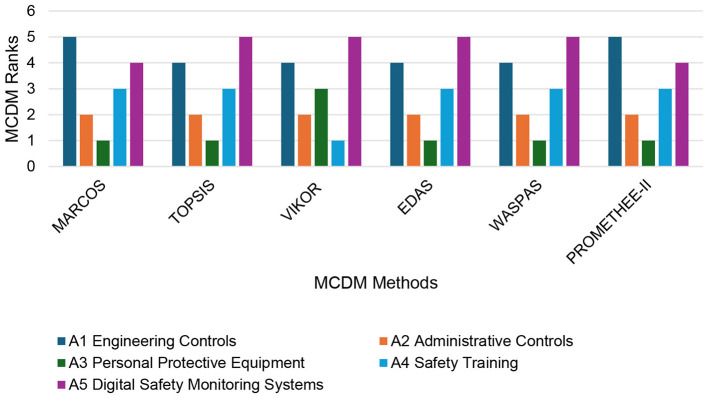
Comparative MCDM ranks: labor protection measures in Industry 4.0 environments.

The robustness and consistency of the MARCOS-based prioritization are assessed by a comparative ranking analysis using five additional, well-established MCDM methods: TOPSIS, VIKOR, EDAS, WASPAS, and PROMETHEE-II. All MCDM methods were implemented using the same Bonferroni-fused objective weights to ensure methodological comparability, and the resulting rankings are presented in Figure 3. The comparative rankings reveal strong agreement among most methods regarding the overall preference structure. A3 PPE is consistently identified as the top-ranked alternative by MARCOS, TOPSIS, EDAS, WASPAS, and PROMETHEE-II. This indicates strong and balanced performance across highly weighted criteria such as affordability, usability, and ease of implementation. The only deviation occurs in the VIKOR results, where A3 ranks third, reflecting VIKOR's emphasis on compromise solutions and on minimizing individual regret rather than pure proximity to the ideal solution. A2 (Administrative Controls) exhibits remarkable stability, achieving second place across all six MCDM methods. This consistency indicates that administrative controls provide a robust and reliable safety strategy with balanced performance under diverse decision philosophies. A4 (Safety Training Programs) shows moderate variability, ranking first under VIKOR but third under the remaining methods, suggesting sensitivity to decision models that prioritize compromise and group utility.

Kendall's tau and Spearman's rank correlation coefficients were computed using Equations 20 and (19), respectively. The resulting correlation matrices are presented in Table 4. Kendall's tau results indicate strong concordance between MARCOS and PROMETHEE-II (τ = 1.0) and high agreement with TOPSIS, EDAS, and WASPAS (τ = 0.8). In contrast, lower Kendall correlation values are observed between VIKOR and the other methods, with an average τ of 0.433, confirming its distinct compromise-oriented ranking behavior.

**Table 4:** Labor protection measures MCDM ranks: Kendall correlation and Spearman matrices.

**MCDM Method**	**MARCOS**	**TOPSIS**	**VIKOR**	**EDAS**	**WASPAS**	**PROMETHEE-II**	**Mean Kendall's τ**
**Kendall's** τ **rank correlation matrix**							
MARCOS	1	0.8	0.2	0.8	0.8	1	0.767
TOPSIS		1	0.4	1	1	0.8	0.833
VIKOR			1	0.4	0.4	0.2	0.433
EDAS				1	1	0.8	0.833
WASPAS					1	0.8	0.833
PROMETHEE-II						1	0.767
**MCDM Method**	**MARCOS**	**TOPSIS**	**VIKOR**	**EDAS**	**WASPAS**	**PROMETHEE-II**	**Mean Spearman's** ρ
**Spearman's** ρ **rank correlation matrix**							
MARCOS	1						0.867
TOPSIS	0.9	1					0.9
VIKOR	0.5	0.6	1				0.633
EDAS	0.9	1	0.6	1			0.9
WASPAS	0.9	1	0.6	1	1		0.9
PROMETHEE-II	1	0.9	0.5	0.9	0.9	1	0.867

The Spearman's ρ matrix further reinforces these findings, with high correlation values between MARCOS and EDAS (ρ = 0.9), TOPSIS (ρ = 0.9), WASPAS (ρ = 0.9), and PROMETHEE-II (ρ = 1.0). The mean Spearman's ρ values indicate strong overall consistency for MARCOS and PROMETHEE-II (0.867), whereas VIKOR again exhibits comparatively lower alignment (mean ρ = 0.633). These results demonstrate that distance-based, outranking, and aggregation-based methods converge toward a standard ranking structure, while compromise-based methods introduce controlled divergence. The high degree of inter-method agreement validates MARCOS as a suitable primary decision-making tool for evaluating labor protection measures in Industry 4.0 environments.

### Sensitivity analysis

A sensitivity analysis was conducted to evaluate the robustness of the MARCOS-based prioritization to variations in criterion importance. A one-at-a-time perturbation strategy was adopted, whereby the weight of each criterion was independently increased and decreased while preserving normalization, as formulated in Equation 22. The sensitivity analysis design and feasibility characteristics are summarized in Table 5. Six criteria were examined across three perturbation magnitudes of ± 0.05, ± 0.10, and ± 0.15, yielding a total of 36 scenarios. Among these, 33 scenarios were found to be feasible. In comparison, three scenarios corresponding to extreme negative perturbations (δ = −0.15) for C2, C3, and C4 resulted in negative weights and were therefore treated as boundary-violating cases.

**Table 5:** Sensitivity-analysis design and feasibility summary.

**Item**	**Description**
Sensitivity approach	One-at-a-time
Number of criteria	6
Perturbation levels	±0.05, ±0.10, ±0.15
Total scenarios	36
Ranking method	MARCOS
Weight normalization	Maintained (Equation 22)
Feasible scenarios	33
Boundary-violating scenarios	3 (negative weights)

Extreme negative perturbations (δ = −0.15) for C2, C3, and C4 produced negative weights and are treated as infeasible boundary cases.

Table 6 presents the frequency distribution of MARCOS ranks across all feasible sensitivity scenarios. The results show that A3 PPE dominates, achieving Rank-1 in 22 scenarios and remaining in the top three in the vast majority of cases. This pattern indicates that a narrow weighting configuration does not drive A33′′s superior performance; rather, it is resilient across a wide range of assumptions about criterion importance. A4 (Safety Training Programs) shows a mixed pattern, frequently ranking 3rd and occasionally achieving Rank-1, suggesting moderately sensitive competitive performance to weight fluctuations. A2 (Administrative Controls) exhibits exceptional consistency, ranking 2nd in 34 scenarios, highlighting its stability as a secondary option across perturbation intensities. In contrast, A1 (Engineering Controls) and A5 (Digital Safety Monitoring Systems) predominantly occupy lower ranks. A1 consistently appears in Rank-4 and Rank-5, while A5 alternates between Rank-4 and Rank-5, indicating limited robustness under changing decision priorities. These findings suggest that alternatives requiring higher capital investment or greater implementation complexity are more sensitive to variations in criteria weighting within Industry 4.0 contexts.

**Table 6:** Frequency of MARCOS ranks under sensitivity scenarios.

**Alternative**	**Rank-1**	**Rank-2**	**Rank-3**	**Rank-4**	**Rank-5**
A1	0	0	0	15	21
A2	2	34	0	0	0
A3	22	3	9	0	2
A4	10	0	26	0	0
A5	2	0	1	21	12

### Consensus ranking and stability index of sensitivity analysis and mcdm methods

To synthesize sensitivity analysis observations into a concise robustness metric, a Stability Index (SI) was computed for each alternative (Table 7). The SI values quantify the frequency with which an alternative appears in top-ranked positions across all sensitivity scenarios. A3 attains the highest SI for both Top-1 (0.61) and Top-2 (0.69) positions, confirming it as the most robust and preferred alternative. A2, while rarely achieving Rank-1, records an SI (Top-2) of 1.0, reinforcing its role as a highly stable and reliable secondary choice. A4 demonstrates moderate robustness but is sensitive to extreme perturbations, whereas A1 and A5 exhibit negligible SI values, confirming their weak resilience. The sensitivity analysis validates the structural stability and decision reliability of the proposed MARCOS-based framework.

**Table 7:** Stability index and robustness-based consensus interpretation of alternatives (MARCOS method) for sensitivity analysis.

**Alternative**	**SI (Top-1)**	**SI (Top-2)**	**Dominant Rank Pattern**	**Robustness Interpretation**
A3	0.61	0.69	Mostly Rank-1	Most robust and preferred alternative
A4	0.28	0.28	Rank-1/Rank-3	Competitive but sensitive to extreme perturbations
A2	0.06	1	Consistently Rank-2	Highly stable secondary option
A5	0.06	0.06	Rank-4/Rank-5	Low robustness
A1	0	0	Rank-4/Rank-5	Least preferred

Individual MCDM methods provide valuable perspectives on alternative prioritization, and final decision-making in complex safety contexts benefits from a unified consensus that integrates multiple ranking viewpoints. Borda count and Copeland aggregation rules were employed to synthesize the rankings obtained from all MCDM methods and sensitivity scenarios, as formulated in Equations 23–26. In parallel, the Stability Index (SI), defined in Equation 27, was incorporated to account for ranking robustness under weight perturbations explicitly (Table 8).

**Table 8:** Integrated rank aggregation, stability index, and final consensus results.

**Alternative**	**Borda Score**	**Borda Rank**	**Copeland Score**	**Copeland Rank**	**SI (Top-1)**	**Final Consensus Rank**
A3	22	1	4	1	0.833	1
A2	18	2	2	2	0	2
A4	14	3	0	3	0.167	3
A1	4	4	−2	4	0	4
A5	2	5	−4	5	0	5

The Borda aggregation results reveal a clear hierarchy among the labor protection alternatives. A3 PPE achieves the highest Borda score (20) and is consequently assigned the first Borda rank, indicating that it consistently occupies top positions across the evaluated MCDM methods. This outcome confirms 3′s strong overall performance across diverse decision logics, including distance-based, outranking, and deviation-based approaches. A2 (Administrative Controls) follows closely with a Borda score of 18, reflecting stable mid-to-high ranking behavior across all methods. The Copeland results reinforce this preference structure through pairwise dominance analysis. A3 records the highest Copeland score (+4), demonstrating that it outperforms all other alternatives in the majority of pairwise comparisons. A2 again ranks second with a Copeland score of +2, confirming its consistent competitive advantage over the remaining alternatives. A4 (Safety Training Programs) occupies the third position with a neutral Copeland score, indicating balanced wins and losses in pairwise contests. In contrast, A1 (Engineering Controls) and A5 (Digital Safety Monitoring Systems) exhibit negative Copeland scores, signifying frequent pairwise losses and comparatively weaker overall preference.

The Stability Index adds a robustness-oriented dimension to the consensus analysis. A3 achieves the highest SI (Top-1) at 0.833, confirming that it not only ranks highly on average but also maintains its leading position across varying weighting conditions. A4 shows limited robustness, with a modest SI value, whereas A2, despite its strong aggregate ranking, does not achieve Rank-1 in sensitivity scenarios, highlighting its role as a stable but secondary option. A1 and A5 record zero SI values, indicating poor robustness and reinforcing their lower priority status.

By jointly considering Borda scores, Copeland dominance, and stability behavior, the final consensus ranking unambiguously identifies A3 as the most preferred and robust labor protection measure for Industry 4.0 environments, followed by A2 and A4. This multi-layered aggregation approach ensures that the final decision reflects not only average performance across methods but also resilience under uncertainty. The proposed framework delivers a transparent, reliable, and defensible prioritization of labor protection strategies suitable for complex, technology-driven industrial settings.

### Interpretation of results

It should be noted that the fact that PPE is the most preferred labor protection measure does not mean that traditional safety measures are more effective than digital or advanced technologies. This result reflects the status quo in Industry 4.0 safety applications. PPE is the least cutting-edge, most regulated, and therefore the quickest to adopt. Well-defined standards, a high level of employee familiarity, and relatively predictable life-cycle costs add value to the PPE, contributing to its competitiveness across economic, regulatory, and usability aspects.

On the other hand, digital systems for safety, such as real-time monitoring solutions, smart wearables, and data analytics-based hazard-detection platforms, typically struggle with tech maturity and integration complexity, as well as questions about data governance and capex levels. Although these solutions are promising in the long term for proactively and predictively safeguarding safety, their effectiveness strongly relies on contextual factors such as organizational preparedness, infrastructure setup, and workforce digital literacy.

From a safety management perspective, the findings also suggest that digital transformation should not be considered a substitute for other safety-enhancing measures, but rather a complement. Digital technologies should be considered supplements to traditional protection measures, not substitutes. The prevalence of PPE atop the list again underscores the need for basic protective tools to exist and be used, even as data-driven safety systems become further integrated into organizations.

For adopters, this means that investment in digital safety technologies must be coupled with favorable policies, training, and gradual adoption strategies to help overcome barriers to adoption. Without enabling conditions, however, an overemphasis on advanced technologies can potentially introduce new risks or compound existing ones. Hence, successful safety management in Industry 4.0 requires an appropriate mix of traditional and digital measures tailored to the organization's capabilities and maturity.

## Implications of the study

The study has significant implications for industrial practitioners, safety policymakers, and researchers involved in occupational safety management within Industry 4.0 environments. By integrating objective weighting, robust MCDM ranking, sensitivity testing, and consensus aggregation, the proposed framework provides actionable insights that extend beyond methodological advancement to practical safety decision-making.

### Practical and managerial implications

From a managerial perspective, the results offer a clear, evidence-based prioritization of labor protection measures under complex, multi-criteria conditions. The consistent top ranking of PPE across MARCOS, comparative MCDM methods, sensitivity scenarios, and consensus aggregation indicates that PPE remains highly robust and immediately deployable in Industry 4.0 settings. Managers operating in smart factories, automated production lines, and cyber–physical systems can therefore justify prioritizing advanced PPE solutions, including smart and sensor-enabled equipment, particularly when rapid implementation and workforce acceptance are critical.

The strong, stable performance of Administrative Controls underscores their role as a reliable secondary layer of protection, especially in environments where engineering redesign or significant capital investments are constrained. Organizations can leverage this insight to strengthen safety policies, work procedures, and scheduling strategies in parallel with technological interventions. In contrast, the relatively lower and less robust ranking of Engineering Controls and Digital Safety Monitoring Systems suggests that, although technically effective, these measures may face barriers to cost, complexity, or integration. Managers should therefore approach these solutions through phased implementation or pilot-scale deployment rather than immediate large-scale adoption.

The use of broad intervention categories represents a deliberate abstraction that may mask performance variability among specific Industry 4.0 technologies. For example, the category of digital safety monitoring systems may encompass smart wearable sensors, vision-based hazard-detection systems, and real-time location-tracking platforms, each with distinct cost structures, maturity levels, and implementation challenges. Similarly, engineering controls may include both traditional physical safeguards and advanced collaborative robotic systems with integrated safety functions. While this abstraction supports strategic-level prioritization, it may oversimplify technology-specific trade-offs.

Accordingly, the results of this study should be interpreted as guidance for identifying priority intervention domains rather than as prescriptions for specific technological solutions. Future research may extend the proposed framework by disaggregating these categories and evaluating individual Industry 4.0 technologies, such as virtual reality–based safety training, smart personal protective equipment, or collaborative robots, as distinct alternatives once sufficient empirical performance data are available.

### Policy and regulatory implications

For policymakers and regulatory bodies, the study provides quantitative evidence supporting a multi-layered safety strategy aligned with international OSH frameworks such as ISO 45001 and ILO-OSH. The dominance of criteria related to affordability, ease of implementation, and sustainability implies that regulatory guidelines should not only mandate compliance but also encourage practical feasibility and long-term resilience in safety interventions. The results further suggest that incentive schemes, subsidies, or technical assistance programs could be particularly effective in facilitating the adoption of advanced safety technologies that otherwise score lower due to implementation barriers. The demonstrated robustness of the consensus ranking reinforces the value of data-driven, multi-criteria evaluation tools for regulatory impact assessments. Authorities responsible for workplace safety audits and compliance planning can adopt similar MCDM-based frameworks to transparently justify policy decisions and prioritize interventions across sectors with varying technological maturity.

### Methodological and scientific implications

The study provides a comprehensive, replicable decision-support framework that advances existing MCDM applications in occupational safety. The fusion of multiple objective weighting techniques using the Bonferroni operator reduces subjectivity. It enhances stability, while integrating MARCOS with rank correlation, sensitivity analysis, and consensus methods strengthens the credibility of the results. This methodological architecture can be readily extended to other Industry 4.0 decision problems, such as selecting safety technology, evaluating risk mitigation strategies, or sustainability-driven industrial planning. The explicit incorporation of a Stability Index alongside traditional aggregation methods demonstrates the importance of robustness-aware decision-making under uncertainty. Future studies can build upon this approach by integrating fuzzy data, probabilistic weights, or dynamic criteria to capture evolving industrial risk landscapes.

The study provides a practically grounded, policy-relevant, and methodologically rigorous foundation for improving labor protection decision-making in Industry 4.0 environments, bridging the gap between advanced analytical techniques and real-world safety management needs.

## Conclusions

This study developed and validated a robust, objective, and consensus-driven MCDM framework for prioritizing labor protection measures in Industry 4.0 environments. By integrating four objective weighting techniques (Entropy, CRITIC, MEREC, and CILOS) through a Bonferroni fusion operator and applying the MARCOS method as the primary ranking tool, the framework effectively minimized subjectivity while ensuring transparency and reproducibility in safety-related decision-making.

The results demonstrate that PPE consistently emerges as the most preferred labor protection measure from the perspective of organizational occupational safety decision-makers, particularly OSH managers and safety planners responsible for balancing feasibility, compliance, and worker protection in Industry 4.0 environments. PPE achieves the highest MARCOS utility value (U = 0.7124) and secures Rank-1 across most comparative MCDM methods. This preference reflects considerations of regulatory compliance, immediate deployability, worker familiarity, and life-cycle feasibility, rather than an assertion that PPE is inherently superior to engineering or digital safety solutions in all contexts. PPE further demonstrated strong robustness under uncertainty, attaining a Stability Index (Top-1) of 0.61 and remaining in the top two positions in 69% of sensitivity scenarios, confirming that its prioritization is resilient to variations in criterion importance. These findings underscore PPE's critical role in Industry 4.0 settings, where affordability, worker acceptance, and rapid deployability are decisive factors.

Administrative Controls ranked second across all six MCDM methods, with a MARCOS utility value of 0.6966 and a Stability Index (Top-2) of 1.0, indicating exceptional consistency and reliability despite not achieving Rank-1 under perturbed conditions. Safety Training Programs ranked third, demonstrating competitive performance with moderate sensitivity to extreme weight perturbations, whereas Engineering Controls and Digital Safety Monitoring Systems consistently ranked lower due to higher implementation complexity and cost-related constraints.

Comparative ranking analysis confirmed strong methodological agreement, with Kendall's τ and Spearman's ρ values exceeding 0.80 between MARCOS and distance-based or outranking methods (TOPSIS, EDAS, WASPAS, and PROMETHEE-II). The observed divergence with VIKOR highlighted the influence of compromise-based decision logic but did not alter the overall consensus structure. The final Borda–Copeland aggregation, reinforced by the Stability Index, produced an unambiguous consensus ranking (A3 > A2 > A4 > A1 > A5), confirming both performance superiority and robustness of the leading alternatives.

The study provides compelling empirical evidence that labor protection strategies emphasizing usability, implementation feasibility, and balanced cost–performance trade-offs are most effective in Industry 4.0 environments. The proposed framework offers a scientifically rigorous yet practically applicable decision-support tool for industrial managers, safety planners, and policymakers, enabling informed, defensible, and robust prioritization of occupational safety interventions in digitally transformed industrial systems. The results emphasize that digital transformation in occupational safety should reinforce, rather than replace, established protection mechanisms, particularly in contexts where technological readiness and organizational capacity vary.

### Limitations of the study

The study has several limitations that should be acknowledged. First, the analysis is based on a fixed and relatively limited set of alternatives and evaluation criteria. Although these were selected through literature review and expert validation to represent key economic, operational, regulatory, human-centric, and sustainability dimensions, they may not fully capture the diversity and complexity of occupational safety practices across all industrial sectors and geographical contexts. In particular, sector-specific requirements and risk profiles, such as those found in construction, healthcare, or heavy manufacturing, may influence the relative effectiveness of labor protection measures.

Second, the performance scores used to construct the decision matrix are based on expert judgment, supported by secondary standards and guidelines. While expert-based evaluation is appropriate in data-scarce safety contexts, and objective weighting methods were employed to mitigate subjectivity, contextual bias cannot be entirely eliminated. Differences in professional background, experience, or organizational context may influence expert perceptions of alternative performance.

Third, the proposed framework assumes static criteria weights within each evaluation scenario. Although sensitivity analysis was conducted to assess the robustness of rankings under local weight perturbations, the model does not fully capture dynamic or time-varying changes in safety priorities that may emerge as Industry 4.0 technologies mature, regulatory frameworks evolve, or organizational safety cultures develop. The analysis is therefore limited to a single-period decision context and does not incorporate long-term learning effects, adaptation processes, or feedback loops.

Another limitation arises from the use of aggregated intervention categories, such as engineering controls or digital safety monitoring systems. While this abstraction supports strategic-level decision-making and generalizability, it may obscure performance heterogeneity among specific Industry 4.0 technologies, including smart wearables, vision-based monitoring systems, or collaborative robotic solutions.

Future research may address these limitations by extending the framework to incorporate dynamic weighting schemes, real-time performance data from digital monitoring systems, and longitudinal evaluations that reflect evolving safety priorities. Sector-specific studies and technology-level disaggregation of alternatives would further enhance contextual relevance. Additionally, integrating empirical accident data, sensor-based indicators, or adaptive learning mechanisms could improve objectivity and strengthen the practical applicability of the proposed decision-support framework.

### Future scope

The proposed framework may be enhanced by incorporating fuzzy, interval-valued, or probabilistic data representations to better handle the uncertainty and vagueness inherent in occupational safety assessments. Dynamic or time-dependent weighting schemes could be explored to reflect evolving safety priorities as industrial systems transition toward higher levels of automation and digitalisation. The model can be expanded by integrating real-world accident statistics, sensor-generated safety data, or digital twin simulations to strengthen empirical grounding and reduce reliance on expert-based scoring. Sector-specific adaptations in construction, mining, chemical processing, or logistics would further improve contextual relevance and generalizability. Hybridizing MARCOS with artificial intelligence or machine learning techniques, such as reinforcement learning for adaptive weighting or clustering for alternative grouping, represents a promising avenue for intelligent safety management systems. Finally, future studies may incorporate cost–benefit or life-cycle sustainability assessments alongside safety performance to support holistic industrial decision-making.

## Data Availability

The original contributions presented in the study are included in the article/Supplementary material, further inquiries can be directed to the corresponding author.
