# Supplementary material for: Decision support framework for prioritizing labor protection measures to enhance workplace safety and compliance in Industry 4.0 environments
Source: Front Public Health. 2026 Feb 24;14:1781020. doi: 10.3389/fpubh.2026.1781020 (PMC12971659; doi:10.3389/fpubh.2026.1781020)
Supplement: Supplementary file 1 [file Table_1.docx]

**Supplementary Material**

**Appendix A. Expert-Based Performance Scoring Procedure**

Performance scores were collected using a structured scoring template distributed to the expert panel. Experts evaluated each labor protection alternative against the defined criteria using a nine-point Likert scale (1 = very low, 9 = very high). Table A1 presents an example of the scoring template used in the evaluation process.

**Table A1: Example Scoring Template**

| **Alternative** | **C1: Life Cycle Cost** | **C2: Effectiveness** | **C3: Regulatory Compliance** | **C4: Acceptance & Usability** | **C5: Sustainability** | **C6: Implementation Feasibility** |
| --- | --- | --- | --- | --- | --- | --- |
| PPE | 1–9 | 1–9 | 1–9 | 1–9 | 1–9 | 1–9 |
